# Reconstructing an ancestral genotype of two hexachlorocyclohexane-degrading *Sphingobium* species using metagenomic sequence data

**DOI:** 10.1038/ismej.2013.153

**Published:** 2013-09-12

**Authors:** Naseer Sangwan, Helianthous Verma, Roshan Kumar, Vivek Negi, Simon Lax, Paramjit Khurana, Jitendra P Khurana, Jack A Gilbert, Rup Lal

**Affiliations:** 1 Department of Zoology, University of Delhi, Delhi, India; 2 Argonne National Laboratory, Argonne, IL, USA; 3 Department of Ecology and Evolution, University of Chicago, 5640 South Ellis Avenue, Chicago, IL, USA; 4 Department of Plant Molecular Biology and Interdisciplinary Centre for Plant Genomics, University of Delhi South Campus, New Delhi, India

**Keywords:** hexachlorocyclohexane, metagenome, pan-genome, last-common ancestor

## Abstract

Over the last 60 years, the use of hexachlorocyclohexane (HCH) as a pesticide has resulted in the production of >4 million tons of HCH waste, which has been dumped in open sinks across the globe. Here, the combination of the genomes of two genetic subspecies (*Sphingobium japonicum* UT26 and *Sphingobium indicum* B90A; isolated from two discrete geographical locations, Japan and India, respectively) capable of degrading HCH, with metagenomic data from an HCH dumpsite (∼450 mg HCH per g soil), enabled the reconstruction and validation of the last-common ancestor (LCA) genotype. Mapping the LCA genotype (3128 genes) to the subspecies genomes demonstrated that >20% of the genes in each subspecies were absent in the LCA. This includes two enzymes from the ‘upper' HCH degradation pathway, suggesting that the ancestor was unable to degrade HCH isomers, but descendants acquired *lin* genes by transposon-mediated lateral gene transfer. In addition, anthranilate and homogentisate degradation traits were found to be strain (selectively retained only by UT26) and environment (absent in the LCA and subspecies, but prevalent in the metagenome) specific, respectively. One draft secondary chromosome, two near complete plasmids and eight complete *lin* transposons were assembled from the metagenomic DNA. Collectively, these results reinforce the elastic nature of the genus *Sphingobium*, and describe the evolutionary acquisition mechanism of a xenobiotic degradation phenotype in response to environmental pollution. This also demonstrates for the first time the use of metagenomic data in ancestral genotype reconstruction, highlighting its potential to provide significant insight into the development of such phenotypes.

## Introduction

Hexachlorocyclohexane (HCH) was one of the most popular pesticides for the control of agricultural pests and vector borne diseases during the latter half of the 20th century, and has subsequently become a key global pollutant culminating in the creation of open sinks with extremely high concentrations of HCH in the soil (for example, ∼450 mg g^−1^) (Jit *et al.*, 2011).

HCH is prepared by the chlorination of benzene in the presence of UV, resulting in the formation of a technical HCH mixture, primarily containing five stable HCH isomers: α (60–70%), β (5–12%), γ (10–12%), δ (6–10%) and ɛ (3–4%) (Lal *et al.*, 2010). Among these, only γ–HCH, also called lindane, has insecticidal properties and purification of one ton of γ–HCH results in 9–12 tons of HCH waste consisting primarily of α- and β-HCH. During the past six decades, ∼600 000 tons of γ-HCH had been generated; yielding 4–7 million tons of HCH waste (Lal *et al.*, 2010). Apart from being carcinogenic, endocrine disruptors and neurotoxins (Kalantzi *et al.*, 2004), these HCH isomers especially β and δ-HCH are highly persistent in the environment (Vega *et al.*, 2007).

The first aerobic bacterium that could degrade lindane (γ-HCH), *Sphingobium japonicum* UT26, was isolated in 1989 from an HCH contaminated upland experimental field in Japan (Senoo and Wada, 1989). This was followed, in 1990, by the isolation of yet another sphingomonad, *S. indicum* B90A, from Indian rice rhizosphere soil repeatedly treated with technical HCH; this strain could degrade even the most recalcitrant HCH-isomer, β-HCH (Sahu *et al.*, 1990). Over the past two decades both UT26 and B90A have served as models to explore the genetics and biochemistry of HCH-isomer degradation (Kumari *et al.*, 2002; Dogra *et al.*, 2004; Suar *et al.*, 2004; Sharma *et al.*, 2006; Nagata *et al.*, 2011), and it is now well established that the degradation of HCH isomers in sphingomonads is mediated through enzymes encoded by *lin* genes (Lal *et al.*, 2010). Among the *lin* genes, *linA*, *linB* and *linC,* which encode HCH dehydrochlorinase (LinA), haloalkane dehalogenase (LinB) and dehydrogenase (LinC), are responsible for the dehydrochlorination, hydroxylation and dehydrogenation of HCH isomers or their intermediates, respectively.

A recent metagenomic survey of a highly contaminated HCH dumpsite revealed (Supplementary Figures S1 and S2) the expected enrichment of *Sphingomonadaceae*, as well as *lin* genes, plasmids, and transposons over a gradient of increasing HCH contamination (Sangwan *et al.*, 2012). To elucidate the ecology and evolution of the HCH-degrading phenotype in this ecosystem, we have reconstructed an *in situ* validated ancestral minimal genotype and provide evidence for the lateral gene transfer of xenobiotic degradative genes under the selective pressure of HCH pollution. This demonstrates for the first time the application of metagenomic data to ancestral genotype reconstruction, and highlights the potential of this approach to elucidate ecological genotype development, including demarcation between environmental and strain-specific traits.

## Materials and methods

### Soil and strain

Soil samples were collected from an open sink of HCH situated at Ummari village, Lucknow, India (27° 00′ 24.7′′ N, 81° 08′ 57.8′′ E) (Jit *et al.*, 2011). As described earlier (Sangwan *et al.*, 2012), subsamples (500 g soil per subsample) were collected at a depth range of 10–20 cm on 17 September 2011. Soil composition, sampling coordinates, methods and physicochemical analysis were performed as explained earlier (Sangwan *et al.*, 2012). Laboratory strain of *Sphingobium indicum* B90A was used for the experiment.

### DNA extraction and sequencing

Total DNA was isolated from each soil subsample (50 subsamples) using the PowerMax Soil DNA Isolation Kit (MO-BIO, Carlsbad, CA, USA). Equal concentrations (5 μg) of the environmental DNA from each subsample was mixed to form a homogenous composite genetic pool. DNA concentrations were quantified using NanoDrop spectrophotometer (NanoDrop Technologies Inc, Wilmington, DE, USA). Cell pellets from 5 ml pure culture of *S. indicum* B90A were subjected to genomic DNA isolation using QIAamp DNA Mini Kit (Qiagen, Venlo, Netherlands). DNA concentrations were quantified using NanoDrop spectrophotometer (NanoDrop Technologies Inc).

Paired-end reads were generated for genomic (3 μg μl^−1^) and metagenomic DNA (100 ng μl^−1^) using the 454 GS FLX Titanium (insert length=2000 bp±10, average read length: 320 bp±49) and the Illumina Genome Analyzer (San Diego, CA, USA) (insert length: 140 bp±6 and average read length, 75 bp±5) platforms, respectively. Illumina raw sequence data were subjected to various quality measures using the Seq-trim (Falgueras *et al.*, 2010) pipeline and pyrosequencing output was analyzed using Roche 454 Analysis software version 2.0 (Branford, CT, USA). Reads below average score Q20, minimum length=50 bp -Illumina and 250 bp -pyrosequencing, and ambiguous bases (including N) were not included in any downstream analysis.

### *De-novo* assembly of genome and metagenome

*S. indicum* B90A genome sequence reads (paired-end) were assembled into contigs using ABySS (Simpson *et al.*, 2009) set at k-mer length of 41. Metagenomic reads (Table 1) were assembled by Metavelvet (Namiki *et al.*, 2012) at k-mer length of 31, insert length=200 bp and expected coverage=auto. BAMBUS-2 (Koren *et al.*, 2011) was used to generate metagenomic scaffolds. Assemblies (genome and metagenome) were validated and coverage was calculated by aligning the raw data (paired-end) back to the contigs using RTG Investigator (http://www.realtimemetagenomics.com). Any consensus mismatch between contig sequence and overlapping reads was considered as misassembly. Contigs from the previous metagenomic survey (Sangwan *et al.*, 2012) were also used (minimum identity=95% and minimum overlap=50%) to bridge gaps between paired-end assembled metagenome contigs from this study.

### Gene calling and annotations

Genes were predicted for *S. indicum* B90A draft genome using Glimmer-3 (Delcher *et al.*, 2007). Metagenome contigs (minimum length=200 bp) were annotated at various hierarchy levels (individual enzymes, protein families, subsystem and cellular processes) using BLASTX (Altschul *et al.*, 1990) (*E*-value=10^−5^) against COG (Tatusov *et al.*, 2001), Pfam (Bateman *et al.*, 2004) and KEGG (Kanehisa *et al.*, 2004) databases. KEGG annotations were used in comparison against previous metagenomic predictions and hierarchical clustering was performed (*n*=1000) on the resultant matrix (sample versus category) using the pvclust package in R (R Development Core team, 2009). To taxonomically characterize and quantify the present genotypes in our metagenome data, individual metagenome reads were mapped over RefSeq (Pruitt *et al.*, 2007) database (release 45, complete microbe) using RTG Investigator (http://www.realtimemetagenomics.com) (sequence identity cutoff=80%). Metagenomic 16S rRNAs were extracted (minimum length ∼150 bp) from contigs using BLASTN (Altschul *et al.*, 1990) and compared against SILVA SSU rRNA database (Pruesse *et al.*, 2007) using RTG investigator. SSU-align (Nawrocki and Eddy, 2010) was used to construct 16S rRNA domain (V3, V4 and V6) specific models corresponding to the bacterial 16S rRNA secondary structures and implementation of these domains in soil metagenome analysis (Vasileiadis *et al.*, 2012). Metagenomic 16S rRNA sequences were aligned against these models and alignments were manually checked for any sequence repetition. Alignment files from RefSeq analysis were processed with SAMtools (Li *et al.*, 2009) and total number of hits per reference sequence was calculated. Mapping results were further quantified into genera specific relative abundance using taxonomical information from their accession numbers. Pseudogenes were predicted in *S. indicum* B90A draft genome using *Psi*-Fi perl script (Guindon and Gascuel, 2003) and by manually checking (minimum identity mismatch=20%) the alignments for frame shift mutations. Pseudogene predictions were validated for sequencing errors by aligning the sequence reads back to the coding sequences (CDSs).

### ANI and tetra nucleotide frequency calculations

Average nucleotide identity (ANI) values were calculated as explained in Konstantinidis and Tiedje (2005). All possible pairwise comparisons were performed between the *S. indicum* B90A draft genome (Anand *et al.*, 2012) and the available *Sphingobium* genomes (chromosomes and plasmids). *S. japonicum* UT26 (Nagata *et al.*, 2010), *S. chlorophenolicum* L-1 (Copley *et al.*, 2012) and *Sphingobium* sp. SYK-6 (Masai *et al.*, 2012) are the reference genomes of the organisms included in this study. Whole-genome-based tetranucleotide correlations were calculated using TETRA (Teeling *et al.*, 2004). Euclidian distance matrix was constructed from ANI and tetranucleotide comparisons and hierarchical clustering was performed on the resultant matrix. These analyses were performed in R (R Development Core team, 2009) using ade (Dray and Dufour, 2007), pvclust and gclus packages.

### Metagenomic recruitment of the genus *Sphingobium*

Metagenomic recruitment plots were generated for all available four genomes (chromosomes and plasmids) of the genus *Sphingobium* with MUMmer (Kurtz *et al.*, 2004). Genomic coordinates covered (sequence identity and query coverage cutoff; 25% and 25% of the region, respectively) by metagenome reads tilling were selected as metagenomic islands (here after described as MGIs) (Steffen *et al.*, 2012). Contigs from this study and previous dumpsite metagenome survey (Sangwan *et al.*, 2012) were reassembled using Minimus (Sommer, 2007) at default parameter. We binned the metagenome contigs corresponding to reference genotypes (genus *Sphingobium*) using tetranucleotide frequencies (correlation cutoff (*R*^2^)=0.9) and %GC criterion (Tyson *et al.*, 2004). BLASTN (Altschul *et al.*, 1990) and MEGAN (Huson *et al.*, 2007) were used to confirm the genetic identities of the contigs. ANI and tetranucleotide correlation values were also calculated for the ‘meta-*Sphingobium'* assembly (contigs) against complete *Sphingobium* reference genomes used in this study.

### Identification and analysis of genomic and MGIs

Following the two step process to predict the complete genomic island profile, SIGI-HMM (Waack *et al.*, 2006) algorithm (sensitivity value=0.7) was used to predict the potential genomic islands in ‘MGI free' (without MGIs) regions of the two genetic subspecies (ANI >98%). Genomic Islands were further annotated (BLASTX; *E*-value=10^−5^) against COG (Tatusov *et al.*, 2001), KEGG (Kanehisa *et al.*, 2004) and a local database created using protein CDSs from all *Sphingobium* genomes used in this study.

### Reconstruction and comparative genomics of the ancestor genotype

To reconstruct the ancestor genotype of HCH-degrading subspecies, we used the complete genomes (chromosomes and plasmids) of *S. indicum* B90A and *S. japonicum* UT26 (ANI value of subspecies level ≥98% and both can degrade HCH isomers). As various *Sphingobium spp.* are known to carry the catabolic genes (*lin* genes) involved in HCH degradation, on their plasmids and secondary chromosomes (Dogra *et al.*, 2004; Nagata *et al.*, 2010; Tabata *et al.*, 2011) we included the same of reference organisms for ancestor genotype estimation. Core genomic regions of *S. indicum* B90A and *S. japonicum* UT26 were separated from genomic islands using coordinates from metagenomic recruitment plots and SIGI-HMM predictions. Homologous anchors were computed in two subspecies using Murasaki (Popendorf *et al.*, 2010) with seed weight-30 and seed length-40. Pre-computed homologous anchors were treated as input to predict orthologous segments using OSfinder algorithm (Hachiya *et al.*, 2009). OSfinder predicts orthologous segments (syntenic) using Markov chain models and machine learning techniques. The advantage of using OSfinder lies in its automatic optimization of parameters to build markov models that increases the accuracy via minimizing the errors usually caused by setting the parameters manually (Pevzner and Tesler, 2003).

Orthologous regions (minimum ancestor genotype) were re-annotated with same strategy as explained above. Hypothetical proteins in the ancestor genotype were compared against the protein database of all available S*phingobium* genomes. MEGABLAST (Zhang *et al.*, 2000) was used to check the homologs of predicted foreign gene sequences (horizontally transferred) in ancestor genotype. Ancestral genotype (gene content) was mapped (BLASTN, *E*-value=10^−10^) against genome sequences of *S. indicum* B90A (Anand *et al.*, 2012), *S. japonicum* UT26 (Nagata *et al.*, 2010) and ‘meta-*Sphingobium*' assembly. Hierarchical clustering (minimum relative abundance=0.8% and s.d. cutoff=0.4%) was also performed on metagenome BLASTX results (Altschul *et al.*, 1990) (*E*-value=10^−5^) analysis against KEGG (Kanehisa *et al.*, 2004) database, using MeV 4.4 (Saeed *et al.*, 2003) with Euclidean distance and Kendall's tau matrices and average linkage clustering.

### Detection of genes under positive selection

Orthologous proteins from *S. indicum* B90A and *S. japonicum* UT26 were subsequently aligned, in a pairwise fashion using the CLUSTAL W algorithm (Thompson *et al.*, 1994). The corresponding nucleotide sequences of these alignments were later aligned, codon by codon, using the pal2nal script (Suyama *et al.*, 2006). Yn00 module of the PAML package (Yang, 2007) was used to calculate the dN/dS ratio for each pair of proteins. For proteins potentially involve in the degradation of phenol, toluene, chlorophenol, anthranilate, homogentisate and HCH, quality processed (quality score cutoff: Q_15_) dumpsite metagenome (pyrosequence data) reads were used to calculate dN/dS ratio using methodology as explained earlier (Tai *et al.*, 2011).

### Graph-based clustering and characterization of repetitive elements in genome and metagenome

Paired-end reads from this study, *S. indicum* B90A genome (insert size=2000±10 bp) dumpsite metagenome (insert size=140±6 bp) and pyrosequence reads from previous metagenome survey (Sangwan *et al.*, 2012) were processed separately for *de-novo* identification and characterization of repetitive elements using RepeatExplorer pipeline (http://galaxy.umbr.cas.cz:8080). Briefly, assembly was performed using CAP3 (Huang and Madan, 1999) program with minimum overlap length for clustering=40% of the length (140 bp for genome and pyrosequence metagenome and 33 bp for Illumina metagenome) and minimum percentage identity criterion was set at 80. Results were analyzed using R program SeqGrapheR (Novak *et al.*, 2010). Graph-based clustering (Novak *et al.*, 2010) predicted 2223 (68% reads used) clusters in the whole genome and 817 870 (19% reads used) clusters in the metagenome. Clusters with similar annotations (minimum 40% percent overlap and 80% identity threshold) were merged and re-analyzed. Contigs (contributing in clusters) were annotated (BLASTX; *E*-value=10^−5^) against a custom database constructed using nucleotide sequences of insertion sequence (IS)-elements, integrases and putative tranposases present in the available genomes from genus *Sphingobium* and INTEGRALL database (Moura *et al.*, 2009). To predict the genetic characteristics of transposon-mediated lateral transfer upon HCH contamination (insert size, expected sequences of insertion sites), we classified the predicted transposons to the ISfinder database (Siguier *et al.*, 2006) using BLASTN (*E*-value=10^−^^10^, minimum identity 80% over 80% query length). Genetic fingerprints of the *de-novo* reconstructed transposons were traced back on the draft assembly of *S. indicum* B90A, the complete genome of *S. japonicum* UT26 and modern lineages using BLASTN (*E*-value=10^−10^).

### Sequence availability

Draft assembly of *S. indicum* B90A is available in Genbank database with accession number AJXQ00000000. Metagenome sequence data have been deposited at DDBJ/EMBL/GenBank under the study accession number of ERP001726 (http://www.ebi.ac.uk/ena/data/view/ERP001726).

## Results and discussion

### Phylogenomics of the genus *Sphingobium*: species to ecotypes

A non-redundant database was created (14.98 Mb, 107 735 reads; clustering cutoff at pan-genome level 97% identity) containing HCH dumpsite metagenomic reads that mapped to four *Sphingobium* genomes. The relative abundance of HCH-degrading genotypes, *S. indicum* B90A and *S. japonicum* UT26, was collectively much higher (91 275 reads; 0.8% of the total metagenome data with ANI >98%) than the non-HCH-degrading genotypes of *S. chlorophenolicum* L-1 and *Sphingobium* sp. SYK-6 (16 460 reads; 0.3% of the total metagenome data with ANI >98%). The four *Sphingobium* species, demonstrated close phylogenetic similarity (16S rRNA nucleotide identity of 97–99.6%) (Pal *et al.*, 2005), but to further establish the evolutionary kinship among these isolates, ANI values were computed with the secondary genetic elements, which often carry the *lin* genes (Nagata *et al.*, 2010; Nagata *et al.*, 2011; Tabata *et al.*, 2011) (Supplementary Figure S3a). Phylogenomic sequence analysis of *S. indicum* B90A and *S. japonicum* UT26 revealed that they are genetic subspecies (ANI=98.04%); whereas *Sphingobium* sp. SYK-6 and *S. chlorophenolicum* L-1 are considerably less closely related (ANI=89%) as previously reported using 16S rRNA gene sequence similarity (Copley *et al.*, 2012). Genetic relatedness was further validated using tetranucleotide profiling, which showed a similar relationship as defined by ANI analysis (Supplementary Figure S3b).

As the abundance of populations closely related to *S. indicum* B90A and *S. japonicum* UT26 (ANI >98%) was high (>0.8%) at the dumpsite, we hypothesized that their close relatives would have inherited a minimal ancestor gene complement. In order to validate this hypothesis, metagenomic sequence fragments were assembled, and the resulting contigs were characterized using tetranucleotide frequency correlation and %GC into *Sphingobium* related bins, which were then reassembled. This ‘meta*-Sphingobium*' assembly was, as expected, genetically (tetranucleotide frequencies and ANI) close to *S. indicum* B90A and *S. japonicum* UT26 (Supplementary Figure S3b). However, mapping the ‘meta*-Sphingobium*' assembly to the non-HCH-degrading *Sphingobium* strains (*Sphingobium chlorophenolicum* L1 and *Sphingobium* sp. SYK-6), clearly highlighted their genetic heterogeneity, corresponding to the higher relative abundance of their secondary genetic elements in comparison with the primary chromosome (Supplementary Figure S3a). The high relative pangenomic abundance (at species level identity cutoff of 97%) of plasmids and secondary chromosome genotypes within the dumpsite metagenome, suggests that both HCH-degrading and non-degrading *Sphingobium* strains have been selected for these secondary genetic elements in comparison with the gene content present on the primary chromosome, which highlights the selection pressure of the HCH contamination.

### Recursive identification of ‘foreign genes'

The individual metagenomic reads (20 111 630 reads) from the dumpsite were mapped on to the draft genome of *S. indicum* B90A, as well as the two chromosomes (chromosome 1=3.5 Mb and chromosome 2=681.8 Kb) and three plasmids (pCHQ1=190.9 Kb, pUT1=31.7 Kb and pUT2=5.3 Kb) of *S. japonicum* UT26 (Supplementary Figure S3a). Regions with >90% sequence identity and a mapping coverage of ∼8 × were selected for ancestor genotype predictions (see next section), and those with low sequence identity (less than ∼25%) or low coverage (less than ∼25% of the region) were characterized as MGIs and further annotated to determine their strain or environment specific functional traits. By using this approach, we identified two MGI profiles; 631 genes (∼312 Kb) in the genome of *S. indicum* B90A and 822 genes (∼559 Kb) in the complete genome sequence of *S. japonicum* UT26.

Although, at a fine-scale (for example, KEGG enzyme) the functional profiles of the two MGIs were not very similar (*R*^2^=0.69 and *P*<0.0001; Fishers two-sided exact test with FDR correction; Supplementary Figure S3c), the majority of the genes still encoded for transposition, recombination and repair (B90A=15.094%±0.13 and UT=11.051%±0.01), as well as inorganic ions and amino-acid transport and metabolism (B90A=10.09%±0.18 and UT26=9.08%±1.4) (Supplementary Figure S3d). As, both the strains have been isolated from different locations (India and Japan), the differences in the functional potential of MGIs profiles could be attributed to the genetic adaptation of these strains in their microhabitats.

Besides the MGIs in both strains using the genomes were further analyzed for foreign genes using DNA composition characteristics via SIGI-HMM (Waack *et al.*, 2006). The existing MGIs for each strain (predicted above) were validated using this algorithm. Although no further foreign genetic material was predicted for *S. japonicum* UT26 (Supplementary Table S2), the genome (excluding the MGI) of *S. indicum* B90A was predicted to contain 51 further foreign protein CDSs, totaling 31 Kb. These genes coded mostly for transposase, phage integrase, recombination and repair, and hypothetical proteins (Supplementary Figure S3d), the majority (89%±0.56) of which had greatest phylogenetic similarity to genes belonging to class Alphaproteobacteria. This demonstrates that metagenomic read mapping combined with DNA composition based algorithms (SIGI-HMM), provide a robust methodology for predicting a ‘complete' genomic island profile.

### Reconstruction of the ancestral genotype and implications for legacy to recalcitrant compound degradation across modern lineages

**Figure 1:**
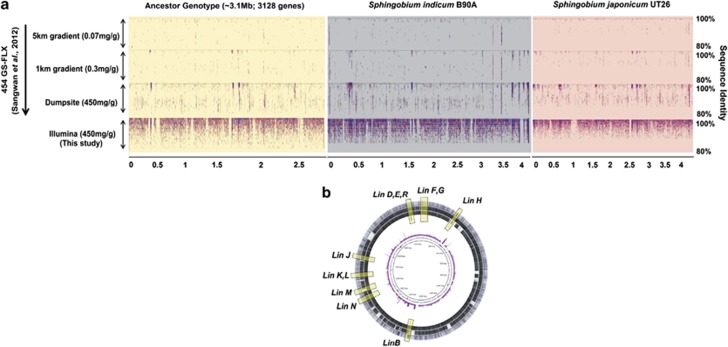
Predicted ancestral genotype, its metagenomic fingerprints and genetic rearrangements across *S. indicum* B90A and *S. japonicum* UT26. (**a**) Metagenomic fingerprints of ancestral gene content; Pyrosequence reads from the previous dumpsite metagenome survey (Sangwan *et al.*, 2012) and illumina reads from this study were plotted on the predicted ancestral gene content (yellow shade) and genome sequences of the *S. indicum* B90A (gray shade) and *S. japonicum* UT26 (pink shade). Each dot on the graph represents an individual sequence read aligned along its reference genotypes. *x* and *y* axis represents the genomic coordinate (Mbp) and percentage sequence identity, respectively. (**b**) Genetic breakpoints between ancestor genotype and HCH-degrading genetic subspecies from outside towards centre; outermost circle: metagenomic contigs, circle 2: draft assembly of *S*. *indicum* B90A, circle 3: complete genome of *S. japonicum* UT26 mapped (BLASTN) over ancestral genotype (black color intensity represent the percentage identity, that is, darker the shade higher is the sequence identity), circle 4: *lin* genes homology between ancestor and subspecies, circle 5: metagenomic read coverage plot of ancestral gene content (5 × as coverage cutoff), outermost circle: GC plot of the predicted ancestral gene content.

The minimal ancestral gene content inherited into each subspecies was reconstructed and its genetic fingerprint traced within the modern lineages. The metagenomic data (Table 1) was mapped (Supplementary Figure S3a) on to the genomes and plasmids of the two abundant subspecies (*S. indicum* B90A and *S. japonicum* UT26), and regions with >90% sequence identity and mapping coverage of >8 × were selected. The analysis was based on the assumption that a gene is ancestral if it was present in orthologous segments of the genetic subspecies (genomic regions without MGIs and SIGI-HMM predictions) and has abundant coverage in the metagenome. In all, a total of 3128 protein CDSs (3.04 Mb) were predicted for the minimal ancestral gene content inherited into sub-species from their immediate ancestor (Figures 1a and b). Putative CDSs from the predicted ancestral genotype were compared and mapped (BLASTN, *E*-value 10^−8^; Figure 1b) against the genomes of *S. japonicum* UT26 and *S. indicum* B90A and the available dumpsite metagenomic sequence data, to determine the relative genetic rearrangements across each discrete subspecies, and to trace the *in situ* relative abundance of these rearrangements in the metagenome (Figure 1b). Strikingly, although the lower HCH degradation pathway (*linD, E, R, F, G,* and *H*) was present in the ancestor, and abundant across all metagenomic data sets, there was a complete absence of two enzymes from the ‘core' upper HCH degradation pathway (*linA*, *C* genes) in the predicted ancestral genotype (Figure 1b). This suggests that the *linA* and *linC* were laterally acquired by both *S. indicum* B90A and *S. japonicum* UT26 (mean pairwise dN/dS >1, Supplementary Figure S4a).

Ecologically homogenous strains showing 1–5% genome-aggregate nucleotide divergence are already known to support the core genome hypothesis (Caro-Quintero and Konstantinidis, 2012) and sequence divergence within their genotypes corresponds to the evolution from the last-common ancestor (LCA). Thus, the fact that two major upper-pathway genes (*linA* and *linC)* of aerobic HCH degradation were not detected in the ancestral genotype, and that the HCH-degrading subspecies (Figure 1a and Supplementary Figure S4b) and the predicted ancestor demonstrate sequence-discrete population status (95–100% sequence similarity), suggests that the lower HCH degradation pathway (*linD, linE* and *linR* genes) is an evolutionary ‘long-lived' event (Caro-Quintero and Konstantinidis, 2012) in this ecosystem. This ‘long-lived' hypothesis is supported by the fact that the lower pathway genes were found in all metagenomic data sets (dumpsite, as well as 1 and 5 km from the dumpsite (Sangwan *et al.*, 2012)), which is potentially explained by the close homology of *linD, linE* and *linR* to other catabolic genes also involved in biodegradation of other recalcitrant chemical compounds and present among several bacterial strains (Copley *et al.*, 2012).

The estimated size of the ancestor genotype is 3 Mb (Figure 1a), which is smaller than that of the two subspecies (*S. indicum* B90A, 4.08 Mb and *S. japonicum* UT26, 4.4 Mb) and the estimated *in situ* bacterial specific average genome size (4.03 Mb±0.17) as predicted from the metagenomic data (Raes *et al.*, 2007). The smaller size of an ancestral genotype has been reported to be a reliable indicator for large-scale lateral gene transfer in bacterial evolution (Dagan and Martin, 2007).

The core-metabolism of the ancestor (Supplementary Figure S4c) was similar to that of the sub-species (complete genomes), but strain-specific functional divergence (Supplementary Figure S4b) was also observed (ancestor versus *S. sphingobium* B90A, *R*^2^=0.99, *P*>0.0001; ancestor versus *S. japonicum* UT26, *R*^2^=0.78, *P*>0.0001). Raw reads from the genome of *S. indicum* B90A, the HCH dumpsite metagenome and the predicted ancestor's CDSs were all mapped on to the recalcitrant compound degradation pathway genes of *S. japonicum* UT26 (phenol/toluene, chlorophenol, anthranilate, homogentisate and HCH) (Nagata *et al.*, 2011) (Supplementary Figure S4b). The data suggest that anthranilate degradation is a strain-specific character for *S. japonicum* UT26, inherited from an immediate ancestor, but completely absent in its genetic sub-species (*S. indicum* B90A) genome and in the *in situ* metagenome.

On the contrary, homogentisate degradation was found to be an environment specific trait (laterally acquired) as it was completely absent in the ancestor genotype and the *S. indicum* B90A (subspecies) genome (Supplementary Figure S5), but was present in the dumpsite metagenome. Sequence mapping analysis (identity >97%) (Supplementary Figure S4b), clearly revealed that phenol and chlorophenol degradation pathway genes were present in the ancestral genotype, the reference species, and their as-of-yet-uncultivated relatives present in the HCH dumpsite metagenome. This suggests the selective maintenance of this trait in the evolution of the sphingomonads exposed to HCH isomers.

As the ‘geographically distant' isolation sites of *S. indicum* B90A and *S. japonicum* UT26 did not share the physicochemical history of the analyzed site except amendment with HCH, these traits appear to be purely ancestral. However, these traits are under strong natural selection pressure (mean pairwise dN/dS >1) and the codon usage patterns are in agreement with the core usage of the reference strains. Following the ‘genome streamlining theory' (Giovannoni *et al.*, 2005) genetic maintenance of these traits at high evolutionary cost still needs to be explored.

### *De-novo* identification and characterization of mobile genetic elements *in situ*

**Figure 2:**
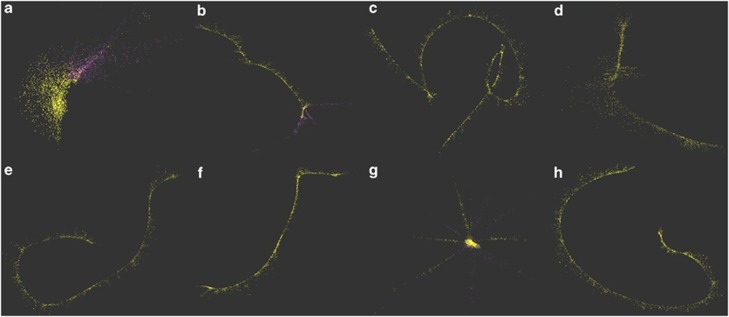
Metagenomic validation of *de-novo* assembled strain-specific mobile genetic elements. Total 8 transposons (**a**–**h**) were selected after reconstructed from *de-novo* assembly and graph-based clustering of genome (*S. indicum* B90A) and HCH dumpsite metagenomes. For each transposon; purple color dots represents the genomic reads of contigs (from graph-based clustering) assigned to a transposon and overlapping yellow reads represents the metagenomic validation with minimum sequence identity of 97%.

The absence of the upper HCH degradation pathway genes *linA* and *linC* in the ancestor generates the hypothesis that the sphingomonads were under strong selection pressure to acquire HCH degradation genes in HCH contaminated environments, and thereby increase their genome size. Therefore, both *S*. *indicum* B90A, *S*. *japonicum* UT26 and the dumpsite metagenome should have the appropriate number of mobile genetic elements, IS and integrases to support these predicted lateral gene transfer events (Aziz *et al.*, 2010). To determine this, a *de-novo* approach was used to identify these elements in the genome of *S. indicum* B90A, and in the HCH dumpsite metagenome. A total of 19 (8.5%) clusters (Supplementary Figure S5a) from the genome and 267 (0.03%) from (Supplementary Figure S5b) the metagenome had significant similarities to the putative transposases and integrases. After merging overlapping clusters (contigs with minimum 40% percent overlap and 80% identity threshold), re-analysis and validation based on the metagenomic coverage cutoff (minimum coverage ∼8 × ), 8 unique clusters (representing complete transposases) were identified (Figure 2). All of the eight putative transposons were found to be from the IS6 family (IS*6100*).

These results suggest that the eight transposons are preferentially environment specific, ecologically selected (1% to 5% pangenomic nucleotide divergence) and highly active (based on their abundance in the metagenomic data) mobile genetic elements and have been enriched in the bacterial lineages present at the dumpsite. Interestingly, on tracing these transposases back to the genomes of *S. indicum* B90A and *S. japonicum* UT26, four of them (Figures 2a) were found to be associated with *linA, linA1, linC* and *linDER* genes, respectively (Dogra *et al.*, 2004; Nagata *et al.*, 2010). These findings clearly explain the strong lateral transfer potential (Tn3 family) of *lin* genes in the reference (genomic) and as-yet-uncultured (metagenomic) HCH-degrading bacterial lineages. It also provides further mechanistic support for the hypothesis that *linA* and *linC* genes are environment specific genetic imports that were not present in the LCA of the two HCH-degrading subspecies.

### Metagenomic recovery of enriched plasmids and a secondary chromosome

**Figure 3:**
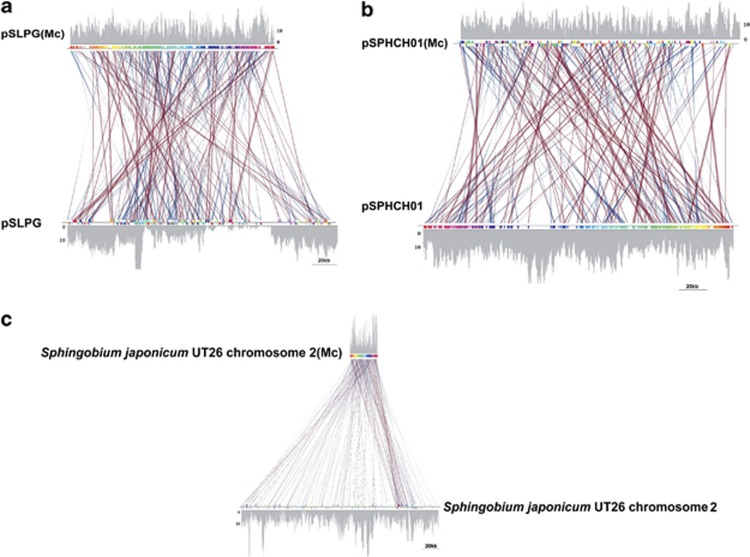
Genotypic synteny and metagenomic recruitment of reference genotypes. Whole-genome alignments between reference genomes and their reconstructed genotypes: (**a**) pSLPG plasmid of *Sphingobium* sp. SYK-6, (**b**) pSPHCH01 plasmid of *Sphingobium chlorophenolicum* L1 and (**c**) chromosome 2 of *S. japonicum* UT26. Overlaid the synteny plots are the metagenomic read coverage on a numerical scale. Mc, metagenome contigs.

Whole-genome sequences of various *Sphingobium* species have revealed that most of the catabolic genes responsible for the degradation of xenobiotics are generally present on their plasmids (Nagata *et al.*, 2011; Tabata *et al.*, 2011; Copley *et al.*, 2012). Comparative metagenomic analysis (BLASTN; *E*-value=10^−8^) against the NCBI plasmid database (ftp.ncbi.nlm.nih.gov/genomes/Plasmids/) revealed the enrichment of plasmids (*P*<0.001 Fisher's exact test with FDR correction) at the dumpsite (Supplementary Figure S5c), and increasing relative abundance across the increasing HCH contamination gradient (5 km <1 km <454 and Illumina dumpsite). For 37 enriched plasmid genotypes (Supplementary Figure S5c), a linear increase (*P*<0.0001) in metagenomic abundance was observed; 19 of which were more abundant in the Illumina data set compared with the 454-pyrosequenced metagenome (Supplementary Figure S5c), potentially as a result of the increased sequencing depth (Supplementary Table S1). Three draft genotypes were assembled from the metagenomic data (Figure 3 and Supplementary Figure S3a) corresponding to plasmids, pSPHCH01 (Copley *et al.*, 2012), pSLGP (Masai *et al.*, 2012), and chromosome 2 of *S. japonicum* UT26 (Lal *et al.*, 2010; Dogra *et al.*, 2004, Nagata *et al.*, 2010). The relative enrichment of previously assembled plasmid variants (plasmid pLB1, pISP3 and pISP4; 12) were also validated. Nucleotide composition- (%GC and tetranucleotide frequency) based clustered contigs (species level structural variants) were arranged using paired end information and reference mapping (minimum percentage identity=85). Relatively similar nucleotide composition patterns were observed in pairwise comparisons between corresponding secondary genetic elements, their primary chromosomes and reconstructed genotypes (Copley *et al.*, 2012).

## Conclusions

The ‘genome streamlining theory' (Giovannoni *et al.*, 2005), posits that selection pressure is linearly correlated with the effective genome size. Here, we have invoked this theory to explain the evolutionary increase in genome size as a result of selective pressure from xenobiotic pollution for microbial lineages present at an HCH dumpsite. Using nucleotide composition patterns, we have reanalysed the genetic relatedness among cultured representatives and *in situ* cohorts of the genus *Sphingobium*, and have shown that use of metagenomic data and combinatorial bioinformatic algorithms can provide a robust methodology for accurate ‘foreign gene' identification. Coherent genetic relatedness within ecotypes and their meta-pangenomic abundance has enabled us to predict the ancestral gene content inherited into their genotypes (strains). Furthermore, this study also proposes that metagenomics has the potential to clearly demarcate between environment and/or strain-specific functional traits acquired by closely related, sequenced reference organisms. These results suggest that we were successful in reconstructing an LCA genotype (as opposed to a core genome) as we were able to (a) clearly demark gene regions that were not part of the two reference genomes, (b) include secondary genetic elements and (c) identify and remove ‘forigen' genes using an LCA-specific algorithm. Strikingly, we found that despite the absence of various xenobiotic chemicals, for example, phenol, toluene, homogentisate and anthranilate, at HCH dumpsite, there is an evolutionary maintenance (from last ancestor to the cultured and yet-uncultivated cohorts) and positive selection of their biodegradation traits. The reasons for this are unclear, but it represents the powerful potential of metagenome-based ancestral genotype reconstruction for uncovering potentially important cross-linked genotype traits in such processes. Further genome sequencing of relevant isolates, and metagenomic sequencing and genome reconstruction from related sites, combined with knock-out mutants can now be targeted towards these processes and genes to help further elucidate the genetic mechanism.

## Figures and Tables

**Table 1:** Sequencing and assembly statistics

*Category*	*Sphingobium indicum B90A*	*Metagenome (Illumina data)*
Sequence data	450 Mbp (320 bp)	1.6 Gbp (75 bp)
Reads after quality filtration	42 044 290	20 111 630
No. of contigs	149 (>500 bp)	1 216 300 (>200 bp)
Average contig coverage^a^	80	3
Reads used in assembly	33 635 432 (80%)	1 608 930(8%)
N50^b^	95 Kbp	253 bp
Max. contig size	∼253 Kbp	∼3 Kbp
GC content	65	61

Abbreviation: N50 is the length of the smallest contig in a bin that contains largest contigs representing at least half of the total assembly length.

a Calculated by mapping of reads to contigs with the criteria of 95% identity over 90% of the read length.

b Based on minimum contig length criteria, that is, genome ⩾500 bp and metagenome ⩾200 bp.
