# Supplementary figures and images for: Reconstructing an ancestral genotype of two hexachlorocyclohexane-degrading Sphingobium species using metagenomic sequence data
Source: ISME J. 2013 Sep 12;8(2):398–408. doi: 10.1038/ismej.2013.153 (PMC3906814; doi:10.1038/ismej.2013.153)

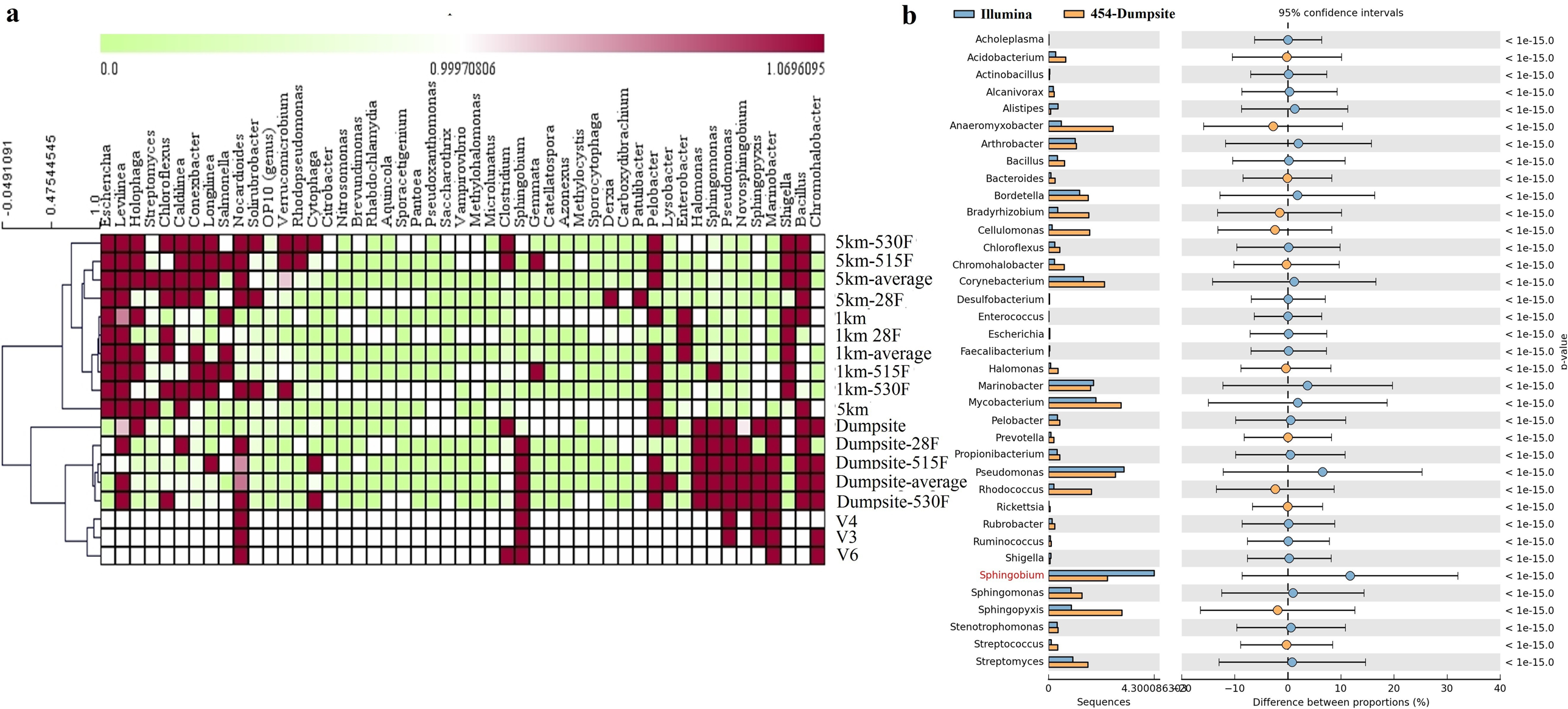

Supplement: Supplementary Figure S1 [file ismej2013153x1.tif]

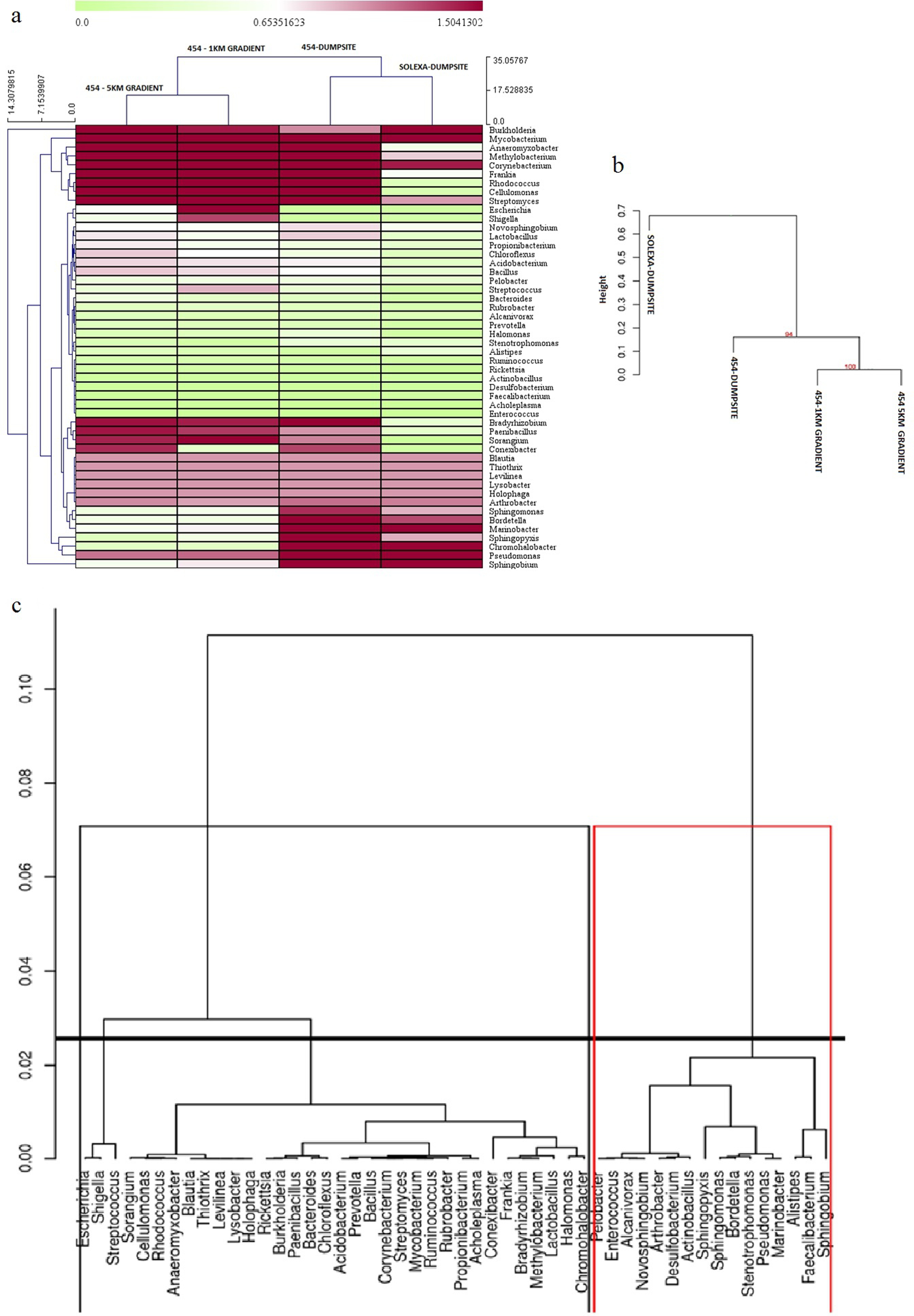

Supplement: Supplementary Figure S2 [file ismej2013153x2.tif]

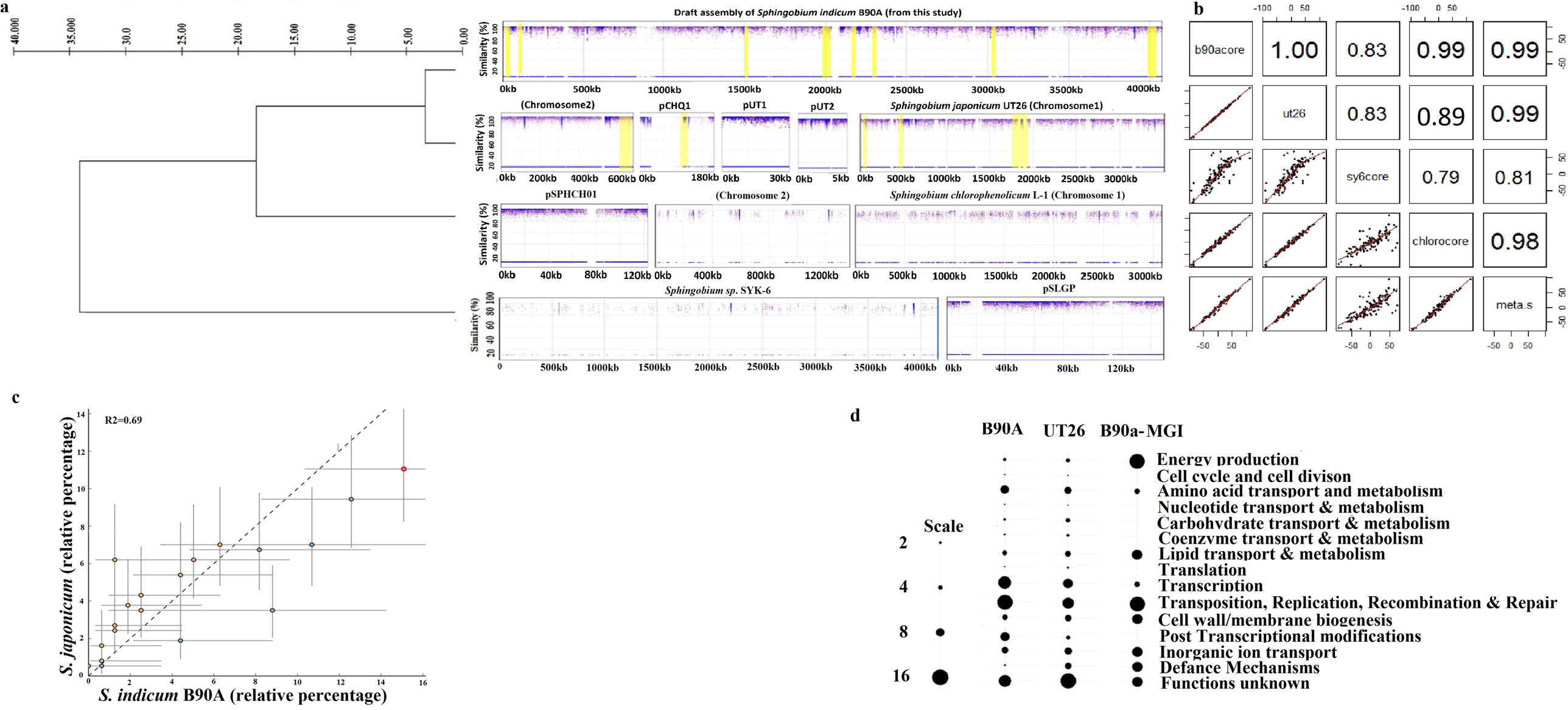

Supplement: Supplementary Figure S3 [file ismej2013153x3.tif]

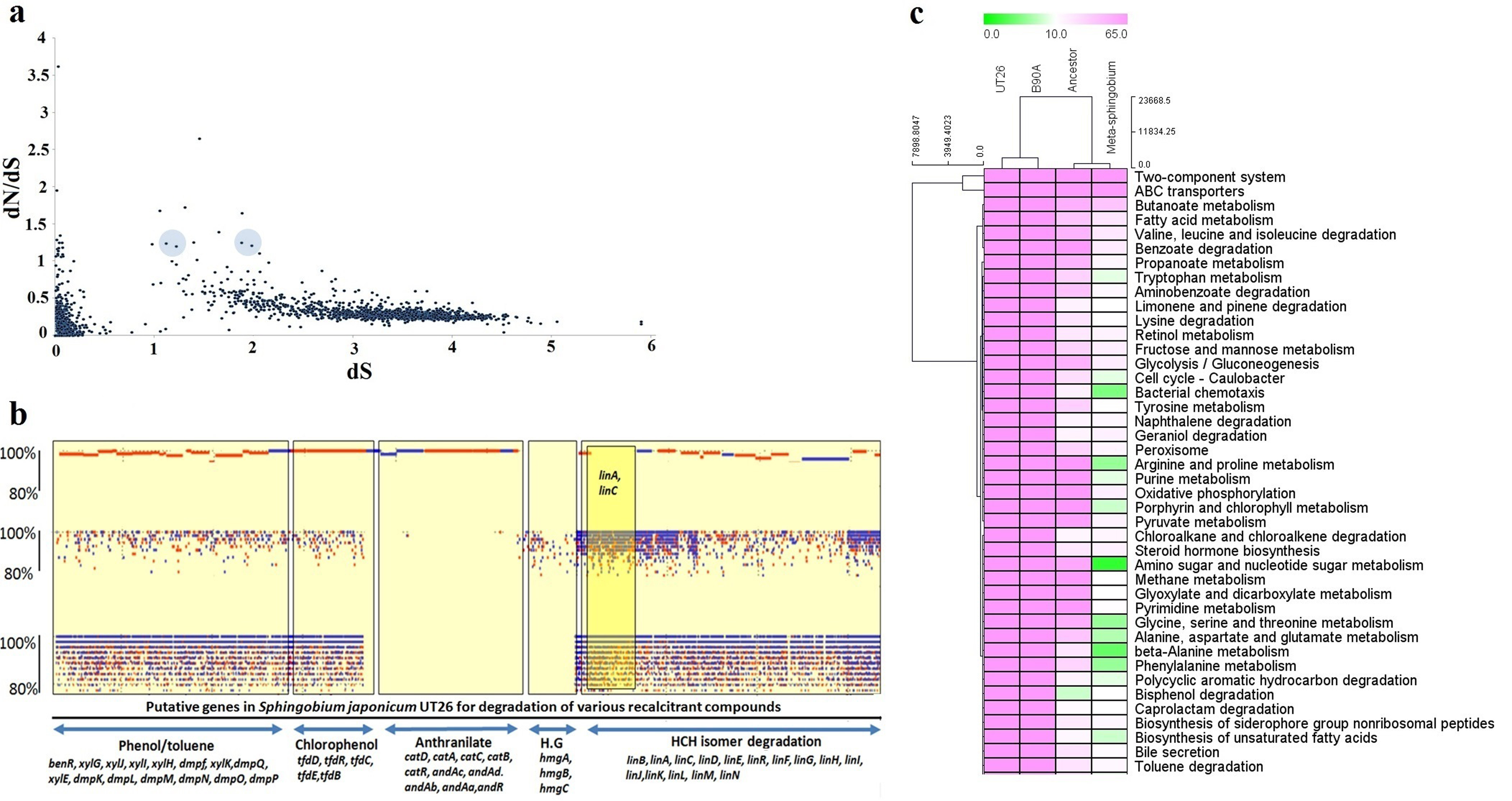

Supplement: Supplementary Figure S4 [file ismej2013153x4.tif]

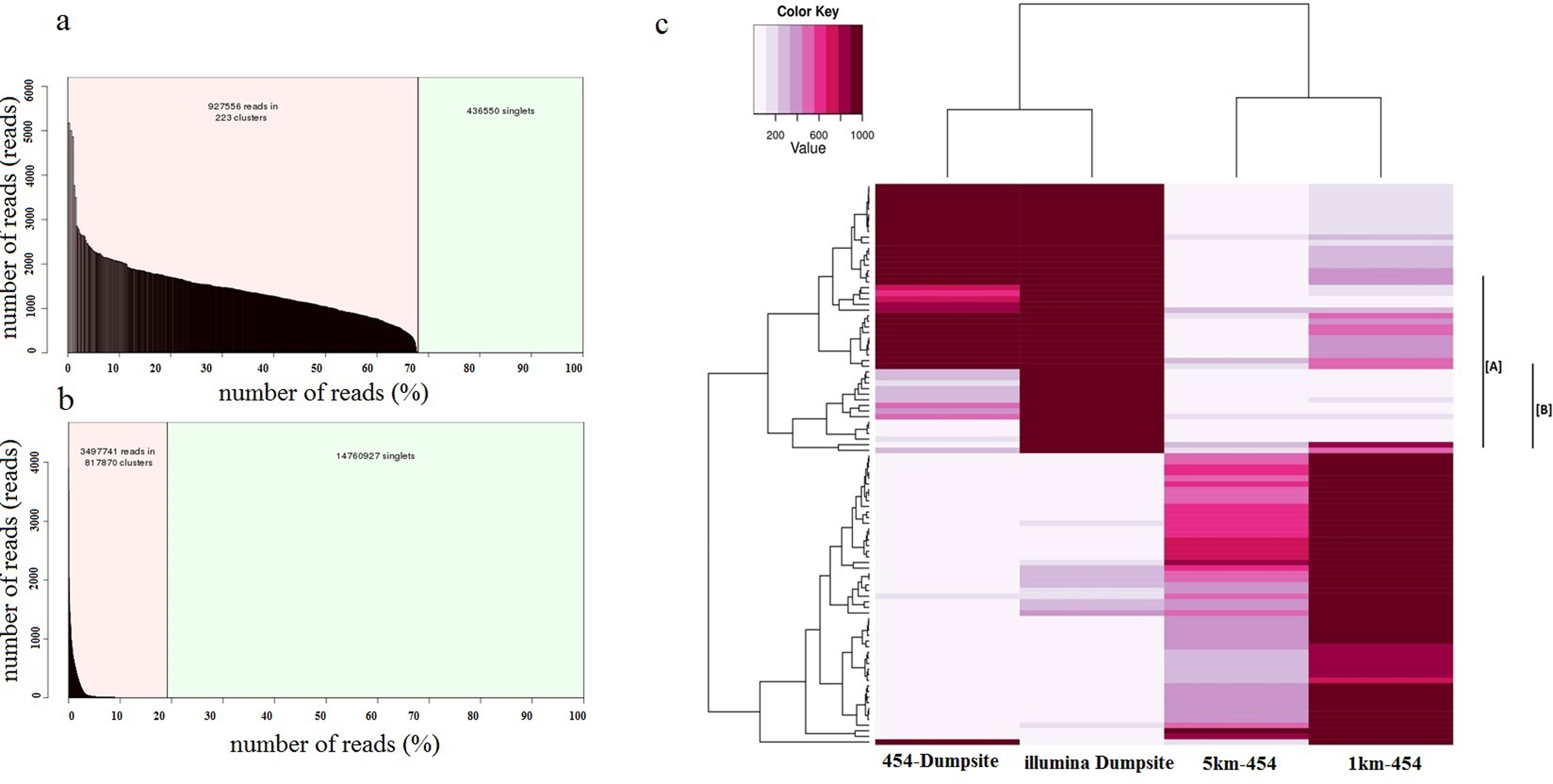

Supplement: Supplementary Figure S5 [file ismej2013153x5.tif]
