# Supplementary material for: Reconstructing an ancestral genotype of two hexachlorocyclohexane-degrading Sphingobium species using metagenomic sequence data
Source: ISME J. 2013 Sep 12;8(2):398–408. doi: 10.1038/ismej.2013.153 (PMC3906814; doi:10.1038/ismej.2013.153)
Supplement: Supplementary Figures Legend [file ismej2013153x6.doc]

**Figure S1** (**a**) 16S rRNA gene based bacterial taxonomy; TEFAP (tag encoded FLX amplicon pyrosequencing) data from Sangwan *et al.,* 2012 and metagenomic 16S rRNA gene sequences from this study (minimum length = 150bp) were compared against SILVA database. Normalised genus versus metagenome matrix was used to perform the two way clustering. Top 50 genera with minimum abundance = 0.8% and minimum standard deviation >0.6 were clustered using Manhattan distance matrix (**b**) Pair wise comparisons (Fisher's exact test with Benjamini and Hochberg False Discovery Rate multiple testing correction) of EGT (environmental gene typing) typing results of dumpsite (Illumina dataset; this study and pyrosequence; Sangwan et al., 2012)

**Figure S2** (**a**) Bacterial diversity patterns at HCH dumpsite; EGT (Environment Gene Mapping) mapping against NCBI RefSeq (n= >15000) database; sample tree was clustered based upon Manhattan distance matrix and average linkage clustering, 50 statistically significant (minimum relative abundance = 0.8% and standard deviation = >0.4) genera have been used for presentation. (**b**) Comparative analysis of community metabolism across HCH contamination; Hierarchical Clustering (bootstrap n =1000, clustering algorithm = average with correlation distance matrix) was performed on the KEGG enzyme annotations (BLASTX ; E-value 10 ^-5^) (**c**) Taxon based enrichment over HCH contamination gradient; Hierarchical clustering was performed on the principal components obtained by ordination (PCA on correlation matrix with 1000 bootstrap) analysis on genus versus metagenome matrix obtained after metagenome 16S rRNA domain specific typing (including TEFAP data from Sangwan *et al.,* 2012) and EGT analysis. Genera with minimum 0.8% relative abundance and standard deviation >0.4% across HCH gradient (Dumpsite illumina = Dumpsite 454 > 1km-dataset > 5km dataset) were selected for clustering and visualization.

**Figure S3** Phylogenomics of the genus *Sphingobium*, and metagenomic fragment recruitment from an HCH dumpsite sample. (**a**) Hierarchical clustering on whole genome based ANI comparisons (measure= distance and clustering algorithm = Un-weighted Average, bootstrap %; n= 1000) and metagenomic recruitment of each genotype is plotted alongside. Purple color dots represent the metagenome reads mapped over reference genome sequence (*X*-axis) with measured percentage sequence identity (*Y*-axis) and yellow color bands represents the genomic coordinates of *lin* genes (**b**) Tetra nucleotide based comparisons of *S*. *japonicum* UT26, *S. indicum* B90A, *S.chlorophenolicum* L-1, *Sphingobium* sp. SYK-6 and Meta-*Sphingobium* assembly. Lower panel represents shows scatter plots of the 256 tetra nucleotide z-scores for each pair wise comparisons and upper panel represents Pearson correlation coefficients. (**c**) Correlation between MGI (Metagenomic Island) predictions of *Sphingobium indicum* B90A and *Sphingobium japonicum* UT26 complete genome. Each Protein coding gene was compared against NCBI COG database and Fishers exact test with FDR correction method was used for statistical significance. (**d**) Each ‘foreign gene’ (putative CDS located on genomic islands) were compared (class level) against NCBI’s COG database and custom database (see material and methods). For *S. indicum* B90A genomic islands predicted from SIGI-HMM were plotted separately.

**Figure** S**4** (**a**) The average dN/dS ratio (*Y* axis) is plotted against the synonymous substitution rates (Ds) (*X* axis). Encircled points represent the genes for which pyrosequence data (Sangwan *et al.,* 2012) of dumpsite metagenome was used for the pair wise dN/dS calculations. (**b**) Strain and/or environmental specific dynamics of recalcitrant compound degradation potential. The *X* axis represents the putative genes from *S.japonicum* UT26 involved in the microbial degradation of phenol/toluene, chlorophenol, anthranilate, homogentisate and hexachlorocyclohexane. Upper panel shows the recruitment of ancestral protein coding sequences, middle panel; individual metagenomic reads and lower panel represents the recruitment of raw sequence from 2kb genomic library of *S.indicum* B90A. Location of *linA, linB* and *linC* genes is highlighted with color gradient. (**c**) Comparative metabolism analysis; ancestor genotype metabolism (KEGG subsystem) was compared against HCH degrading subspecies and metagenome contigs corresponding to *Sphingobium* genomes (binned out using tetra-ESOM and % GC). Two way clustering was performed using with Euclidean distance and Kendall’s tau matrices and average linkage clustering.

**Figure S5** (**a**) Graph based clustering of *Sphingobium indicum* B90A genome; paired end reads were assembled using cap3 program and clustering was performed with minimum overlap length = 40% of the length (140bp) and minimum percentage identity criterion was set at 80. (**b**) Graph based clustering of HCH dumpsite metagenome; paired end reads were assembled using cap3 program and clustering was performed with minimum overlap length = 40% of the length (33bp) and minimum percentage identity criterion was set at 80. (**c**) Plasmid genotype enrichment over HCH contamination: Metagenomic reads were compared against NCBI plasmid database. Normalised (read assigned to a genome/total reads assigned against database). Euclidean and Manhattan distance matrix were used for the two way clustering. [A] Represents the genotypes significantly enriched (*P* value < 0.0001 for all pair wise comparisons) over HCH contamination (Dumpsite > 1km > 5km). [B] represents genotypes enriched (*P* value < 0.0001 for all pair wise comparisons) in Dumpsite-illumina dataset. Names and relative abundance of all the genera used in the generation of this heat map is provided in the Supplementary file 8.
