# Supplementary material for: Reconstructing an ancestral genotype of two hexachlorocyclohexane-degrading Sphingobium species using metagenomic sequence data
Source: ISME J. 2013 Sep 12;8(2):398–408. doi: 10.1038/ismej.2013.153 (PMC3906814; doi:10.1038/ismej.2013.153)
Supplement: Supplementary Table S1 [file ismej2013153x7.doc]

**Table S1**. Sequencing and assembly statistics. A subset of this data is given in the Table1.

|  | ***Sphingobium indicum* B90A** | **Metagenome-Sequence** | | | |
| --- | --- | --- | --- | --- | --- |
|  | **(illumina)** | **Dumpsite (illumina)** | **Dumpsite**  **(454)** | **1 Km**  **(454)** | **5 Km**  **(454)** |
| Sequence data | 450 Mbp (75bp) | 1.6 Gbp (75bp) | 401 Mbp | 383 Mbp | 400 Mbp |
| Reads after quality filtration | 42044290 | 20111630 | 1187505 | 1124891 | 1187505 |
| No. of contigs | 149 | 1216300 | N. D | N. D | N. D |
| Avg. Contig coverage | 80X | 3X | N. D | N. D | N. D |
| Reads used in assembly | 80% | 8% (1,665,161) | N. D | N. D | N. D |
| N50 | 95Kb | 253bp | 700 | N. D | N. D |
| Max. Contig Size | ~253Kb | 3Kb | ~3 Kb | N. D | N. D |
| GC content | 65 | 61 | 62 | 60 | 60 |
