# Supplementary material for: Reconstructing an ancestral genotype of two hexachlorocyclohexane-degrading Sphingobium species using metagenomic sequence data
Source: ISME J. 2013 Sep 12;8(2):398–408. doi: 10.1038/ismej.2013.153 (PMC3906814; doi:10.1038/ismej.2013.153)
Supplement: Supplementary Table S2 [file ismej2013153x8.doc]

**Table S2.** Foreign gene prediction by SIGI-HMM algorithm.

| **SIGI-HMM**  **Predictions** | ***Sphingobium indicum* B90A** | ***Sphingobium indicum* B90A**  **without**  **MGI** | ***Sphingobium japonicum* UT26** | ***Sphingobium japonicum* UT26**  **without**  **MGI** |
| --- | --- | --- | --- | --- |
|  |  |  |  |  |
| Normal | 83.9% | 90% | 81% | 100% |
| CDS | 4423 | 3792 | 4059 | 3237 |
| Putative foreign genes | 631 | 51 | 822 | 0 |
| t-RNA | 54 | N.D | 56 | N.D |
| r-RNA | 7 | 7 | 9 | 9 |

ABBREVATION: **MGI;** Metagenomic Islands
